# Online psychotherapy as a first clinical experience during the Covid-19 pandemic: A new generation of psychotherapists in the digital age

**DOI:** 10.1016/j.heliyon.2024.e29464

**Published:** 2024-04-09

**Authors:** Birgitta Schiller, Martin Kuska, Stella Becher-Urbaniak, Eva Wimmer, Manfred Reisinger, Kathrin Mörtl

**Affiliations:** a Institute for Qualitative Research, Sigmund Freud Private University Vienna, Austria; b Faculty of Psychotherapy Science, Sigmund Freud Private University Vienna, Austria; c Research Department University Outpatient Clinic for Adults, Sigmund Freud Private University Vienna, Austria

**Keywords:** Psychotherapy research, Digital healthcare, Body in psychotherapy, Training research, Qualitative research

## Abstract

The temporary closure of the Outpatient Psychotherapy Clinic at the Sigmund Freud Private University in Vienna during the Covid-19 pandemic demanded an immediate and unexpected reaction to assure further psychotherapeutic services. Both psychotherapists and patients were forced into a rapid transition to online psychotherapy. While Covid-19 research has comprehensively described challenges of online psychotherapies, we were interested in learning specifically how early stage psychotherapists-in-training, who started their clinical work with patients exclusively in the online setting, experienced this unprecedented clinical situation.

Sixteen in-depth interviews were conducted with psychotherapists in training. The data were analyzed using a thematic analysis. The analysis revealed how psychotherapists in training were able to cultivate a set of early-training resources and competencies in the online therapy setting without evidence-based guidelines from supervisors and the institution. This study highlighted the necessity of incorporating specific and novel educational input that is necessary for achieving specific online skills in the early training phase. Recognizing that the therapeutic landscape has undergone an irreversible transformation, the data suggest that distinct techniques are necessary to equip early-training psychotherapists for the now commonly practiced alternation between online setting and in-person setting in psychotherapeutic processes.

## 1 Introduction

The sudden shift to online therapy in the spring of 2020 due to the lockdown measures adopted in response to the Covid-19 pandemic was a massive challenge for the entire field of psychotherapy worldwide. Although the impact of the Covid-19 pandemic on helping professionals-in-training has been the focus of many recent studies, the interest in psychotherapists in training remains largely a peripheral part of larger quantitative studies [[1], [2], [3], [4], [5], [6], [7], [8], [9], [10], [11]]. What can generally be said is that therapists were overwhelmed and thus felt insecure in their professional role and clinical work [[12], [13], [14], [15], [16], [17]]. Everyone, including psychotherapists, was influenced by this experience in their private and professional lives [[18], [19], [20], [21], [22], [23]]. Implementing the abrupt switch to online channels that had barely been used before meant dealing with new challenges, such as technical utilization, and with new psychological phenomena, such as Zoom fatigue [12,[24], [25], [26], [27]].

Our qualitative study captures a historically unprecedented situation in which psychotherapists in training suddenly found themselves unprepared to start their early training experience with patients. All of their training input had been focused on how to deliver psychotherapy face-to-face. Although online psychotherapy before Covid-19 was common in some European countries (e.g. Greece) providing psychotherapy through the online setting was not allowed in Austria. The psychotherapy law simply did not recognize the therapeutic work with patients via online tools as psychotherapy. Thus, preparing students for the online setting was not part of the curriculum [28,29]. Psychotherapists in training who had already started their clinical work face-to-face could assort to their face-to-face experiences with their patients in order to adapt and transfer their skills. We were interested in a specific group of psychotherapists in training, namely those who find themselves in a vulnerable phase in their training: after three years of basic theoretical training and first clinical supervised internship experiences, students will start seeing their own patients in a one-on-one setting. This training phase yields specific challenges for psychotherapists in training and needs to be accompanied by a supportive and mindful supervision system. Studies have shown that psychotherapists who are at the beginning of their professional development have increased levels of anxiety [[30], [31], [32]]. Uncertainty and doubt are typical in this stage of professional education and were now combined with the anxiety-evoking challenges of the pandemic [33]. Sigmund Freud Private University (SFU) offers academic training in psychotherapy science, and psychotherapists in training who start their clinical practice with patients in the clinic are usually 24 years old and have no previous experience in working with psychotherapy patients one-on-one. Psychotherapists in training who are younger can start training until they reach the age of 24. Psychotherapists in training need to spend at least the first 100 sessions of therapy work at a clinic before they can choose to continue working with their patients in their private practices or stay at a clinic for a longer time.

Our study provides rather unique reflections of psychotherapists in training who had their first contact with patients online and who had to develop their own set of skills how to establish a valid alliance. Alliance building in the first set of sessions is crucial for good psychotherapy outcomes [34]. Their expert supervisors may have collected their own first experiences in the online setting but there were no clinical or evidence-based guidelines available how to deal with challenges or early ruptures in the online setting. Additionally, these therapist-patient couples had to transfer an exclusively online-built psychotherapeutic relationship to a psychotherapeutic relationship face-to-face. This was novel in an early training situation. Thus, our aim in this study was to investigate specifically (1) which challenges early-training psychotherapists in training did experience in the online setting, (2) how early-training psychotherapists in training achieved certain specific online competencies, and (3) how they experienced transferring an online psychotherapeutic relationship to the face-to-face setting in later stages of the psychotherapeutic process.

## 2 Method: thematic analysis about psychotherapists in training

The presented study is part of a collaboration of the research team of the University Outpatient Clinic for Adults and the Institute for Qualitative Psychotherapy Research at the Sigmund Freud Private University (SFU) in Vienna. Sixteen semi-structured interviews [35] were conducted with psychotherapists in training during the fall of 2021. The selection criteria were a) psychotherapists in training enrolled in the psychotherapy program at the time, b) psychotherapists in training who had engaged in online therapy sessions during the Covid-19 pandemic, including telephone and videophone modalities and c) a diverse range of psychotherapists in training with varying levels of experience and exposure to online therapy to ensure a comprehensive understanding of the phenomenon.

Each interview lasted from 20 to 50 min and was audio-recorded with participants' consent. An ethic approval was given trough the Sigmund Freud Private University Ethics Board. The audio recordings of the interviews were transcribed verbatim, ensuring accuracy, and preserving participants' verbal expressions.

The primary focus of our overarching research project was the exploration of the significance of the body in the online therapeutic landscape, with prior findings of the grounded theory methodology disseminated in an edited volume “Therapy+” [36]. Acknowledging the unique context of psychotherapists in training gaining initial clinical experience online, our research team undertook a nuanced examination of this facet. Building upon the extensive dataset from the initial grounded theory study, we conducted a secondary analysis, employing a rigorous thematic analysis [35]. This secondary analysis is presented here.

In the initial phase, interviews underwent meticulous re-encoding, culminating in a comprehensive thematic summarization. The identification of themes involved summarizing and categorizing these codes into overarching thematic areas. This process underwent multiple checks and validations through regular team meetings with the involved researchers (Students of the research internship and research coordinator of the University Outpatient Clinic, senior researchers and some research assistants of the Institute for Qualitative Psychotherapy Research), where emerging concepts were discussed, potential interpretations illuminated, and possible biases identified. This not only facilitated ongoing validation but also provided room for the expansion and elaboration of the emerging category system.

Care was taken to ensure that this iterative approach maintained the consistency and reliability of the thematic analysis. Any uncertainties or discrepancies in the data were resolved through open discussions within the research team, thereby ensuring the integrity of the interpretations. The methodology adhered to established standards, including those proposed by Lincoln and Guba (1985) [37], Mayring (2007) [38], Charmaz, and Thornberg (2021) [39], to strengthen the credibility of the results.

### 2.1 Participants

Participants were therapists in training at the SFU University Outpatient Clinic in Vienna. The inclusion criteria were:

- Therapists in training enrolled in a psychotherapy science program.
- Participants who had engaged in online therapy sessions during the Covid-19 pandemic, including telephone and videotelephone modalities.

### 2.2 Sampling

For our exploratory study, we adopted a purposeful sampling strategy, a method commonly employed in the initial stages of Grounded Theory studies [40]. Our sampling criteria were designed to encompass therapists in training who possessed (1) prior experience conducting therapy in-person before transitioning to online platforms, and (2) therapists in training who commenced their therapeutic practice exclusively online during the Covid-19 pandemic. This dual approach allowed us to capture diverse perspectives: therapists with experience in both traditional and digital settings (1) offered unique insights into the nuances and challenges of delivering therapy across different mediums, while those who started their practice online during the pandemic (2) provided valuable insights into the process of adapting to a digital environment. Understanding how therapists navigate and succeed in building therapeutic relationships and delivering effective interventions solely online contributes significantly to our understanding of online psychotherapy.

In our study, it became apparent that interviews with therapists in training from diverse age groups and practicing various psychotherapeutic modalities were necessary to gain a comprehensive understanding of the adaptation processes. Therefore, we tailored our sampling approach accordingly. This theoretical sampling aimed to broaden the understanding of online psychotherapy by encompassing a wide array of experiences and perspectives within the specific context of therapists' age and therapeutic orientations [41,42].

### 2.3 Participant characteristics

The sampling methodology employed in this study ensured the inclusion of a varied and inclusive cohort of therapists in training, encompassing crucial sociodemographic and modality-related attributes. Through this approach, the study captured a rich tapestry of experiences and viewpoints among participants, considering factors such as gender (No other gender identities were present in our sample, than indicated in Table 1.), age, therapeutic approach, and living arrangements. These diverse perspectives provide valuable insights into both the efficacy and hurdles associated with online psychotherapy practice.

**Table 1:** Sample characteristics.

	**Gender**	**Age group**	**Psycho-therapeutic modalities**	**Spatial possibilities for online therapy**	**Living Situation**
Interview 1	Female	40–49	Systemic therapy	Own practice room	With 5 other people
Interview 2	Female	40–49	Individual psychology	Room at home	With one other person
Interview 3	Male	30–39	Individual psychology	At home	Living alone
Interview 4	Male	20–29	Individual psychology	At home	Living alone
Interview 5	Female	20–29	Individual psychology	At home and own practice room	With one other person
Interview 6	Female	20–29	Psychoanalysis	At home and own practice room	Living alone
Interview 7	Female	20–29	Psychoanalysis	At home	Living alone
Interview 8	Female	20–29	Person-centered therapy	At home	With 2 other people
Interview 9	Female	30–39	Gestalt	At home	Living alone
Interview 10	Male	20–29	Person-centered therapy	At home	With 3 other people
Interview 11	Female	20–29	Behavioural therapy	not specified	Living alone
Interview 12	Male	30–39	Gestalt therapy	At home	With one other person
Interview 13	Female	20–29	Systemic therapy	Room at home	With 3 other people
Interview 14	Female	30–39	Systemic therapy	Own practice room	With 4 other people
Interview 15	Female	20–29	Gestalt therapy	Room at home	With one other person
Interview 16	Male	50–59	Gestalt therapy	Own practice room	not specified

## 3 Results

### 3.1 Lost space and framework – online space has no institutional structure

Most psychotherapists in training starting their clinical practice do not have their own private practice space. The interviewed psychotherapists in training have not yet finished their studies and, furthermore, they have the obligation to conduct the first 100 sessions of their clinical one-on-one practice with patients at the SFU University Outpatient Clinic. Many of these novice psychotherapists in training had ideas and expectations about the clinic's premises, but hardly any of them had even visited or appropriated the site of the clinic when the Covid-19 pandemic broke out. Until the government decided on pandemic regulations, there was a great lack of certainty about whether therapies could take place at all, whether the clinic must close immediately, or if switching to an online setting was even legally possible. In a matter of only a couple of days the psychotherapists in training had to decide and inform their current and new clients whether they would meet at all, whether they could see them on site at the clinic, whether they were to be provided an alternative private practice room, or whether they would switch to the online setting, and if so, on which platform. Switching to the online setting came with specific challenges concerning an improvised therapy room: some psychotherapists in training do not live alone. They share their apartments with roommates, have pets or live with their partner or family members. Therefore, finding a neutral, undisturbed space with a good Internet connection was challenging. Strategies for providing a safe and exclusive therapy space for psychotherapists in training varied widely. Some asked their cohabitants to leave the apartment for a while and, for example, to go for a walk. However, there was nowhere to go during the first lockdown; some had large “do not disturb” warning signs pinned to their doors. When possible, spaces other than the bedroom were used for the purpose of therapy. If there was no shared-space room or designated home-office space in the apartment, the psychotherapists in training looked for external locations. These were not particularly practice-appropriate either, such as an old dance studio, a vacant apartment or even the abandoned premises of the university. For those who decided to conduct online therapy at home the issue of the separation between private space and time and workspace and time became evident:

*“I also don’t have a practice where I can say this is a space for myself for these purposes, where I could withdraw and go there as a psychotherapist. Therefore, I did it from home, which was difficult. This separation - am I a private individual now, am I a professional. Getting dressed and reading through the protocols from the last sessions was almost like a ritual before the session.”* (Interview 10) The switch to the home office also meant that there were no routes anylonger to be travelled for going to and leaving the SFU University Outpatient Clinic. Therefore, entering or exiting the inner psychotherapeutic space had to happen in a different mode. The experience of how this felt physically was not yet manifested in the body, and the correct ratio between private and professional space had to be found.

On the one hand, there were no more set guidelines and structures from the institution; on the other hand, there was also no encouragement and support, neither from the clinic director or experienced psychotherapists and supervisors who give advice and support on-site, nor from colleagues who communicate their own newly gained experiences or offer a brief door-to-door talk. During the lockdown, it was possible to make use of supervision online, but all the informal and in-between conversations got lost. Discussing experiences from therapy with roommates, partners or parents was not permitted, and if it did happen, it had to be distorted and anonymized, so that it was more likely to cause frustration because those involved could not (fully) relate to the reality of starting a clinical career.

Usually, the appropriation process of the psychotherapeutic role is very natural and unconscious for the psychotherapists in training. Through the narration, it became clear how much the premises and, in this case, the institution of the SFU University Outpatient Clinic missed out on giving appropriate guidance for this adaptation process, and professionalization had to be produced experimentally.

### 3.2 Appropriation of the psychotherapeutic habitus

It could be shown in the analysis that safe spaces are essential in appropriating the professional role. However, not only the room itself, with its physical entry and exit, but also other factors that enable a change of role on various levels are essential. Equally important is a person's appearance and how others perceive one as a psychotherapist. While in training, psychotherapists dress and style themselves differently when they are with their peers, and their appearance and habitus change almost naturally as they enter the clinical profession. What otherwise happens very subtly became a deliberate ritualized action during the lockdown. The psychotherapists in training spoke of how much focus they put on dressing professionally and thoughtfully before a session. Other professionals working in a home office in front of a screen may have gone without official clothing in non-visible areas. This was not an option for the aspiring psychotherapists in training, because they had to consciously put themselves into a professional state. At most, it was possible to be barefoot at times or cross-legged, which they would never do in the practice room. Nevertheless, the psychotherapists in training needed to have a good and comfortable seat and maintain an upright sitting position to avoid falling into a too relaxed mood.

*“I don’t actually sit there in my pajamas or anything; I still sit in my work clothes. On the phone, I just sit somehow comfortably, I do that already.”* (Interview 14)

In addition, meticulous care was taken to remain within a professional framework visually. The background of an online session was also used to reinforce the required seriousness, preferably by creating a white wall.

*“I made sure that behind me everything is white, that you could not perceive any element of my apartment at all, which was important to me, simply because I wanted to show myself as protected as possible. And yes, that did something with me: I took my clients into my home.”* (Interview 5) Likewise, the quality of lighting was controlled, as was the perception of the opposite party by considering even the color of the clothing in any given interaction.

Appointments for therapy sessions could be more freely scheduled because of the time saved by the lack of travel to and from the Outpatient Clinic and due to there being no restrictions on booking an institutional room. With this gained freedom, sessions were arranged at varying and unusual times. If a single session was assigned on a Friday evening, psychotherapists in training changed their clothes only for this 1 h. Mixing the clinical with the private space was inconceivable in both directions. Nothing personal should intrude into the work setting, nor should anything clinical spill over into the private sphere. Thus, it was difficult for some psychotherapists in training to leave the psychotherapeutic role immediately, and it sometimes caused an unpleasant feeling after a therapeutic conversation, e.g., when naked in the shower afterwards, as one interviewee reported. For others, it was easier to separate the two spheres, and all it took was to close the laptop to be back in the private space. However, if they could not manage to get into the professional role, everything seemed like a game.

*“I experience myself much more alive in [*face-to-face*] therapy, I can concentrate better; yes, it is insanely fun. I've only had the first few sessions with my clients, and it's made a huge difference for me. Honestly, it didn’t feel as real that I was now working as a psychotherapist, because I was doing it from my living room and didn’t go to the Outpatient Clinic or have to deal with other psychotherapists. So, for me, it’s more real now.”* (Interview 9)

There is no model to learn from, no mirror, and no confirmation. No institution provides the framework, and there are no conversations with peers to ensure everything is all right. The psychotherapeutic experience is still a novel experience. Doing all this on their own creates even more doubts than the usual uncertainty at the beginning of clinical work.

The psychotherapists in training managed this step, which otherwise happens almost by itself, through very conscious actions and reflection. But the containment and the mindful creation of their own clinical space was a unique challenge for the psychotherapist beginners.

### 3.3 Closeness and distance in online setting, reduced embodiment and the surprise at the first meeting in person

One of the most discussed topics in the online therapy literature is that of presence [[43], [44], [45], [46], [47], [48]]. This refers to the feeling of being present together as resonance generates a particular moment in the present, and a relationship can be built. The state of being present is predominantly produced by the body and is almost tangible for the participants. If it cannot be created, the conversation remains superficial, the participants’ shared perceptions separated by a third medium, the online space. Some of the psychotherapists in training already had previous experience in clinical face-to-face practice and were able to transfer existing patients into online therapy. They know how their clients move within the space, what dynamics arises in the dialogue and what signals their body sends. The creation of presence in a spatial separation, only with the image of the upper half of the body transferred via a laptop, is still complex but easier to establish with this background knowledge.

*“What is not perceived is simply everything that happens below the upper body, so to speak, whether someone is now fidgeting with their feet and is nervous or with their hands or something like that. What is also missing and is not perceived is the way someone walks, stands, enters the room, shakes hands, or how someone greets you. If you look at someone, if you look away, there is an uncomfortable atmosphere right from the start that is missing and is not perceived. But I think that everything that is showing on the face, the facial expressions, is perceived right away.”* (Interview 7)

Some psychotherapists in training had never had a session in a face-to-face setting before starting online therapy, and they first met their patients online or even by phone. Through the interviews, this circumstance provided great insight into the feeling of presence and the play between closeness and distance. It was reported that clients were sometimes perceived much differently online than when they were seen in person. With direct contact, it was like getting to know the person anew. Many described that although a therapeutic relationship could be established and they worked successfully, additional information gave a deeper insight into who the person is afterwards. One factor in this is the attitude and presentation in the online setting. One client, for example, leaned over the cell phone from above and thus appeared very active. At the clinic in a face-to-face setting, on the other hand, she sank into her chair, and it became clear that there was hardly any energy in her. Another patient seemed restless in presence and shifted positions in her seat, which was not noticeable online.

*“The facial expressions I could perceive well, which is now difficult to work out at the Outpatient Clinic with the masks on. I do not know what is better. But the physical aspect, the posture – I have seen a client for the first time again at the Outpatient Clinic, the first time in my presence, and have noticed that she is extremely restless. She mentioned that herself at the end that she was constantly changing her posture and was very restless, and of course, I can pick up on that well, and I honestly didn’t notice that at all, although she also did that at home.”* (Interview 9)

Many of the psychotherapists in training were surprised when they first got to know their clients in person and had to revise some of their hypotheses about them. Some clients were not visible or hardly visible in the online sessions because the use of technical means was overwhelming for them. Some sessions were only conducted by telephone, provoking various imagined ideas about looks and other physical aspects. However, all these circumstances were also beneficial in a therapeutic sense, as long as they could be brought into the therapy as material and be discussed. The psychotherapists in training also report being extremely active in the online sessions and addressing more topics than in the face-to-face sessions. The senses had to be sharpened, so nuances in speech and voice pitch were accurately distinguished to determine what was happening with the other person. Most uncomfortable were the pauses where the physical separation was barely tenable — e.g., not being able to reach out to the other person, especially in crises, sometimes triggered strong reactions.

*“I had an experience at the very beginning where I thought to myself – shit – why online – I didn't know the client very well, and she slipped into a crisis on-screen, and I wasn’t there, and that made me feel uneasy, I honestly have to say. I could intervene in a completely different way on-site compared to when she escalates on-screen and breaks down crying, and I can no longer see her on the screen, and I don’t know what is happening on the other side. […] Believe it or not, I went in with a certain attitude and tone of voice and managed to calm the patient down.”* (Interview 16)

The fantasy of losing one's opposite mostly turned into the realization that by verbalizing it, it was possible to contain the patient. More than usual, feelings were localized in the psychotherapists' in training own body and also inquired about in the patients. If no possibility of access could be found, the conversations remained on a level of “talking heads.” The mental catharsis of the clients was increasingly exhausting for some psychotherapists in training. Others could realize the difference only within the face-to-face setting and sometimes used online sessions to protect themselves during the first hours of clinical work.

Psychotherapists in training had not yet sufficiently learned and refined their therapeutic style and potential. In the invisible space, not only patients but also psychotherapists in training could hide at the beginning of their profession. However, as indicated in the interview, it is only fun if you are fully engaged.

*“It [face-to-face therapy] gets under your skin more, gets to you more, and has more direct access to you. And if you’re not used to that, it’s suddenly overwhelming. […] I am not a fan of the online setting simply for these reasons. I think the work is limited by it. As a psychotherapist, you must get involved with all the feelings that come along when you are in the same room, with this intimacy, with this privacy, with the closeness also being present. I think that only then does psychotherapy become efficient. And if you can do it well, I think, and by now, I think I can do it a little better. Also, only then is it enjoyable.”* (Interview 4)

Ambivalences permeate online therapy, and the advantages can simultaneously be disadvantages. Therefore, it depends on the attitude towards online therapy. Although the psychotherapists in training had to deal with the absence of direct and close physical contact in their first clinical work experiences, their sensitivity was trained and enriched to the extent which they would not have experienced in any other setting.

*“I can say a lot of things now only based on the contrast with online therapy, because it was only then that I noticed a lot of things that were so natural before.”* (Interview 3)

### 3.4 Everywhere, every time, everything: the ambivalence of flexibility

At first, the flexibility of online psychotherapy seemed to be an enrichment for many psychotherapists in training, and only later did they realize that it may also bring possible challenges. Many of those who started at the SFU University Outpatient Clinic were no longer guided by the fixed schedules of a regular practice routine. With the gained time, they took on more patients and arranged appointments in irregular or unorthodox ways. The status of always being available had to be questioned later on, because its continuation back at the clinic is impossible. Reflections about enactment and the realization of having to be more consistent arose.

*“But precisely this increased flexibility, these processes also affect this dissolution of the boundaries of work. This increased flexibility led me to think to myself, ‘Oh, go on, we’re in a good process* right *now, so we’ll add another hour so that it will work out [ …].’ There I was in a bit of a gray area with my time resources.”* (Interview 3)

But the allocation of appointments is not the only area where the psychotherapeutic field was delimited. The space itself became flexible. Suddenly, it was possible for psychotherapists in training and clients to attend sessions from anywhere. Patients living in remote regions were accepted, and the psychotherapists in training themselves retreated to, e.g., the countryside. Therapy sessions were held in cars, on balconies, in the garden or during a walk in the park. It was possible to go abroad, either for a vacation or for professional reasons. Most psychotherapists in training still had part-time jobs besides their training. Injuries or physical disabilities were not a problem, as long as presence could be established. Everything seemed easily compatible, without considering that both lockdown and obligatory online therapy would eventually end. It was demanding to process parallel events and at times difficult for the psychotherapists in training to switch into the psychotherapeutic role quickly when, for example, the laundry was done in between sessions, the dog needed a walk or lunch was prepared.

*“Now I live with my partner and my dog in a one-room apartment, which means we always had to coordinate very well because of data protection. It just doesn’t work that he’s there when I’m working*. *We also have a balcony, but when it rains, it’s stupid. I now have another practice room I’m renovating, so I can go there with my laptop to work. That is, I also have the journey to and from, because I go by car to this room, but before that …, I think that the boundaries are a bit more blurred, if you do it in your own home and then just have a quick coffee and then quickly go out with the dog during the break.”* (Interview 6)

Nevertheless, the issue of flexibility remains highly ambivalent. In contrast, there was a tenor of looking forward to face-to-face therapy and appreciating the benefits of structure, even if it meant losing certain freedoms tied to body and space.

## 4 Discussion

Through an analysis of the experiences of the psychotherapists in training it becomes apparent how important it is for the formation of the professional psychotherapeutic role to have a designated space, a set framework and an accustomed structure to adopt the habitus of the common field [49,50]. Further studies have shown that the body (and embodiment), in combination with space, plays an enormous role in online therapy [[51], [52], [53]]. Despite the challenging situation, the hurdles were still successfully overcome by reflection and the creation of ritualized behavior, as well as increased conscious self-care. Adapting to the new conditions, not only that of professional practice, but additionally, the dissolution of boundaries in the online space, required an increased expenditure of energy [18,30,33,54]. This effort enabled the acquisition of new and otherwise rarely experienced skills that would not have been possible in the regular setting.

The psychotherapists in training developed increased sensitivity to sensory perceptions and abilities. They gained better awareness of the body (their own and the clients’). They experienced that physicality is a crucial part when it comes to assuming the role of a psychotherapist. Many otherwise unrecognized actions could be reflected upon; unconscious dynamics provoked by the online setting could thus be made conscious. All these resources had a benefit and could be used for further clinical work.

Presence is a well-known topic in the discussion of online therapy. The body perceptions are necessary for establishing and maintaining a psychotherapeutic relationship [[43], [44], [45], [46], [47], [48]]. Recent studies conducted during or after the Covid-19 pandemic indicate a physical limitation in online therapy which presents a significant challenge in contrast to face-to-face therapy [19,25,53,55,56]. Moving away from the comparison and recognizing online therapy as a setting in its own right allows the conclusion of a new psychotherapeutic technique. In this sense, it is no longer about advantages or disadvantages but the different requirements and possibilities [17,19,55,[57], [58], [59], [60], [61], [62], [63], [64]]. One requirement is the increased awareness of the body to compensate for missing components. If this is successful, online therapy represents an opportunity for psychotherapeutic care, especially in a digital era [16,17,36,52,[65], [66], [67], [68]].

The psychotherapists in training were often relieved to return to the institution of the SFU University Outpatient Clinic. They looked forward to meeting clients in person, exchanging ideas with colleagues and having some time to engage with their newfound role.

These findings are consistent with previous research considering the various circular phases and processes of psychotherapy training [[30], [31], [32], [33],^69^,^70^]. There is a massive uncertainty at the beginning of a professional training that requires space, structure and a foothold.

Nevertheless, re-entering the “old systems” was also experienced as rigid, which creates a paradoxical situation. Psychotherapists in training experienced flexibility in the same way as their patients, which now, it seemed, had to be abandoned again. Psychotherapy has changed irrevocably with the global shift, and the psychotherapists in training have experienced a psychotherapeutic space that they will not be able to discard [36,[71], [72], [73], [74]]. Nor will they become acquainted with the pre-existing therapeutic field, as their experience set has already been shaped from the beginning by the perceptions of online therapy. A generation that has grown up with the technical know-how and digital networks of the modern world, knowing the potential in clinical work, cannot be denied further exposure to online therapy [8,33]. The media age has already begun for psychotherapy [65,71,72,75,76]. For psychotherapists in training at the start of their career, in particular, this was not an upheaval but normality from the start, and they are now transferring it into their further practice. This circumstance makes it necessary to continue to investigate the field of online therapy, especially the importance of the body in the digital space. Setting training priorities and adapting method-specific techniques adequately for online treatment is now a requirement [8,27,33,77]. Sensitizing psychotherapists in training to the indications and possibilities of using online therapy as a targeted intervention is no longer a goal for the future, especially when the current generation of psychotherapists in training and the majority of clients have already arrived and are in the midst of it.

## 5 Research limitations and outlook

The findings presented herein stem from a secondary analysis. Moving forward, it is imperative to conduct further research, particularly in the realm of training, to integrate online therapy seamlessly into psychotherapeutic education. Moreover, extensive investigations are necessary to address ethical considerations and adapt legal frameworks. This encompasses the development of curricula that equip therapists with the requisite skills for this digital era, ensuring they are professionally adept in this evolving landscape.

## Ethics statement

Ethic approval received on the 9th of April 2021 by the Ethic Commission of the Faculty for Psychotherapy Science of the Sigmund Freud Private University Vienna.

Check digit and internal digital signature of the university: FBZNQ32EAYMP2188590.

## Data availability statement

The data associated with our work has not been deposited into a publicly available repository. The data will be available on request.

## CRediT authorship contribution statement

**Birgitta Schiller:** Writing – original draft, Formal analysis, Data curation, Conceptualization. **Martin Kuska:** Writing – review & editing, Supervision, Resources. **Stella Becher-Urbaniak:** Writing – review & editing, Validation, Project administration, Formal analysis, Data curation. **Eva Wimmer:** Writing – review & editing, Methodology. **Manfred Reisinger:** Writing – review & editing, Validation. **Kathrin Mörtl:** Writing – review & editing, Validation, Supervision, Conceptualization.

## Declaration of competing interest

The authors declare that they have no known competing financial interests or personal relationships that could have appeared to influence the work reported in this paper.
